# Supplementary material for: Photoactive Pore Matrix for In Situ Delivery of a Photosensitizer in Vascular Smooth Muscle Cells Selective PDT
Source: Materials (Basel). 2019 Dec 9;12(24):4110. doi: 10.3390/ma12244110 (PMC6947284; doi:10.3390/ma12244110)
Supplement: Supplementary file 1 [file materials-12-04110-s001.pdf]

materials

Article

Photoactive Pore Matrix for In Situ Delivery of a Photosensitizer in Vascular Smooth Muscle Cells Selective PDT

Magdalena Wawrzyńska [1], Maciej Duda [2], Iwona Hołowacz [2], Aleksandra Kaczorowska [2], Agnieszka Ulatowska-Jarża [2], Igor Buzalewicz [2], Wojciech Kałas [3], Edyta Wysokińska [3], Dariusz Biały [4], Halina Podbielska [2] and Marta Kopaczyńska [2,*]

1 Department of Emergency Medical Service, Wroclaw Medical University, Parkowa 34, 51-616 Wroclaw, Poland; mag.wawrzynska@gmail.com
2 Department of Biomedical Engineering, Wroclaw University of Science and Technology, Wybrzeze Wyspianskiego 27, 50-370 Wroclaw, Poland; maciej.duda@pwr.edu.pl (M.D.); iwona.holowacz@pwr.edu.pl (I.H.); aleksandra.kaczorowska@pwr.edu.pl (A.K.); agnieszka.ulatowska-jarza@pwr.edu.pl (A.U.-J.); igor.buzalewicz@pwr.edu.pl (I.B.); halina.podbielska@pwr.edu.pl (H.P.)
3 Department of Experimental Oncology, Ludwik Hirszfeld Institute of Immunology and Experimental Therapy, Polish Academy of Sciences, ul. Rudolfa Weigla 12, 53-114 Wroclaw, Poland; wojciech.kalas@hirszfeld.pl (W.K.); edyta.wysokinska@hirszfeld.pl (E.W.)
4 Department and Clinic of Cardiology, Wroclaw Medical University, Borowska 213, 50-556 Wrocław, Poland; dariusz.bialy@umed.wroc.pl
* Correspondence: marta.kopaczynska@pwr.edu.pl; Tel.: +48-71-320-46-17

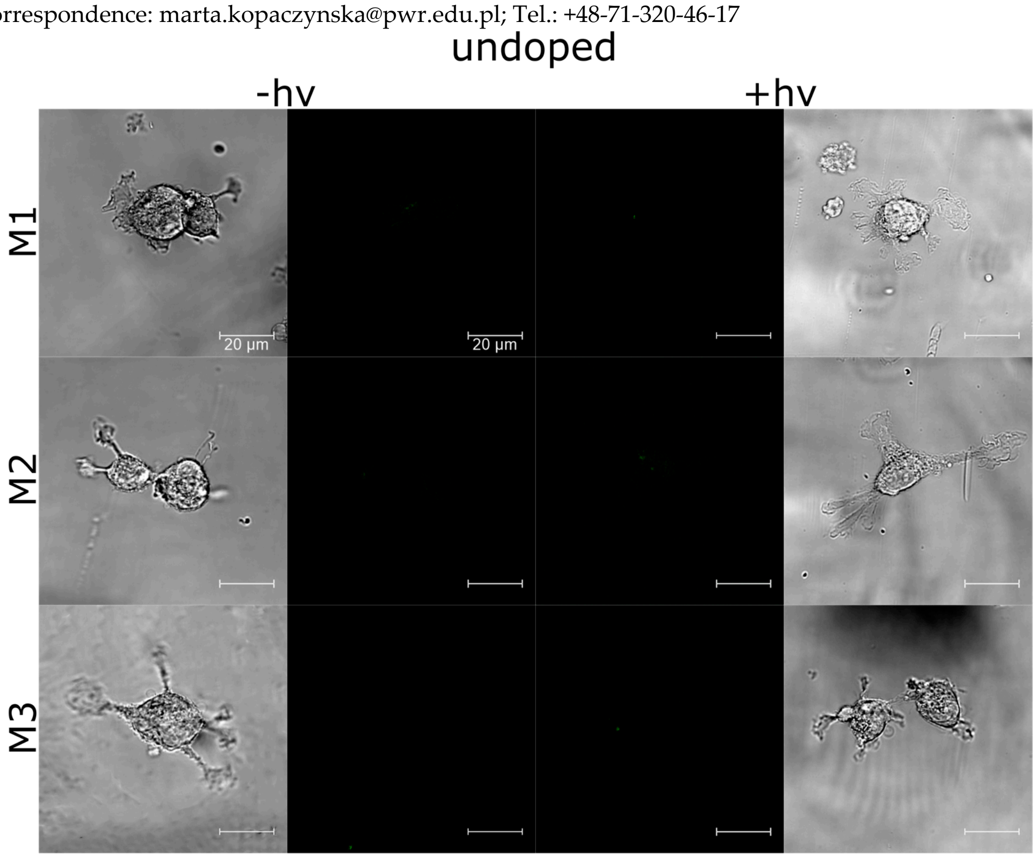

Figure S1. Confocal and bright field images of HUVEC incubated on undoped sol–gel layers M1, M2, and M3 before (left panel) and after (right panel) PDT. Cells on undoped surfaces do not exhibit fluorescence.

© 2019 by the authors. Submitted for possible open access publication under the terms and conditions of the Creative Commons Attribution (CC BY) license (http://creativecommons.org/licenses/by/4.0/).
